# Caveolin-1 regulates chemokine receptor 5-mediated contribution of bone marrow-derived cells to dermal fibrosis

**DOI:** 10.3389/fphar.2014.00140

**Published:** 2014-06-11

**Authors:** Rebecca Lee, Beth Perry, Jonathan Heywood, Charles Reese, Michael Bonner, Corey M. Hatfield, Richard M. Silver, Richard P. Visconti, Stanley Hoffman, Elena Tourkina

**Affiliations:** ^1^Division of Rheumatology and Immunology, Department of Medicine, Medical University of South CarolinaCharleston, SC, USA; ^2^Department of Regenerative Medicine and Cell Biology, Medical University of South CarolinaCharleston, SC, USA

**Keywords:** bleomycin, monocyte, migration, fibrocyte, adipocyte, scleroderma, lipodystrophy

## Abstract

In fibrotic diseases caveolin-1 underexpression in fibroblasts results in collagen overexpression and in monocytes leads to hypermigration. These profibrotic behaviors are blocked by the caveolin-1 scaffolding domain peptide (CSD) which compensates for caveolin-1 deficiency. Monocytes and fibroblasts are related in that monocytes are the progenitors of fibrocytes (CD45+/Collagen I+ cells) that, in turn, are the progenitors of many fibroblasts in fibrotic tissues. In an additional anti-fibrotic activity, CSD blocks monocyte differentiation into fibrocytes. We studied a mouse fibrosis model (Pump Model) involving systemic bleomycin delivery that closely models scleroderma (SSc) in several ways, the most important of which for this study is that fibrosis is observed in the lungs, skin, and internal organs. We show here that dermal thickness is increased 2-fold in the Pump Model and that this effect is almost completely blocked by CSD (*p* < 0.001). Concomitantly, the subcutaneous fat layer becomes >80% thinner. This effect is also blocked by CSD (*p* < 0.001). Even in mice receiving vehicle instead of bleomycin, CSD increases the thickness of the fat layer. To study the mechanisms of action of bleomycin and CSD, we examined the accumulation of the chemokine receptor CCR5 and its ligands MIP1α and MIP1β in fibrotic tissue and their roles in monocyte migration. Fibrocytes and other leukocytes expressing CCR5 and its ligands were present at high levels in the fibrotic dermis of SSc patients and Pump Model mice while CSD blocked their accumulation in mouse dermis. Migration toward CCR5 ligands of SSc monocytes and Pump Model bone marrow cells was 3-fold greater than cells from control subjects. This enhanced migration was almost completely blocked by CSD. These results suggest that low monocyte caveolin-1 promotes fibrosis by enhancing the recruitment of fibrocytes and their progenitors into affected tissue.

## Introduction

Skin is usually the most involved organ in systemic sclerosis (scleroderma, SSc). Fibrotic lesions characterized by thickened dermis, enlarged follicles, and obliterated blood vessels initially involve the reticular dermis, then spread to the adjacent adipose layer and ultimately to internal organs (Altman et al., 1991; Silver, 1995). Diffuse cutaneous SSc describes the most severe form of the skin disease which is particularly likely to spread to internal organs and result in death (Abraham and Varga, 2005).

While historically it was believed that fibrosis results from the activation by cytokines of resident fibroblasts into myofibroblasts that overexpress collagen, recent studies suggest that fibroblasts can also be derived from other cell types including: (1) Bone marrow-derived cells, (2) Epithelial cells via epithelial-mesenchymal transformation, and (3) Pericytes (Hung et al., 2013). We are particularly interested in the bone marrow lineage in which monocytes differentiate into fibrocytes (recognized by their co-expression of hematopoietic markers such as CD45 and mesenchymal markers such as collagen (I) which in turn differentiate into fibroblasts. Fibrocytes or their precursors are recruited into target tissues by chemokines present at high concentration that create a chemotactic gradient. The ability of cells to sense the chemotactic gradient depends on the presence of appropriate chemokine receptors on the cell surface. In addition to their role as precursors of fibroblasts, fibrocytes also participate in fibrosis by releasing a variety of cytokines and chemokines (e.g., TGF-β, MCP-1, MIP1α, MIP1β) (Chesney et al., 1998) and angiogenic factors (e.g., VEGF, PDGF-A, and IL-1β) (Hartlapp et al., 2001). It is noteworthy that fibrocytes secrete chemokines that recruit additional fibrocytes into fibrotic tissue.

Observations on multiple cell types including fibroblasts and monocytes frompatients and from animal models have suggested that a common feature of fibrotic diseases is the underexpression of caveolin-1, a protein that acts as a master regulator of several signaling pathways by binding to and thereby inhibiting the function of multiple kinases (Tourkina et al., 2005, 2008, 2010, 2011; Wang et al., 2006). Likewise, the skin and lungs of caveolin-1 null mice are fibrotic (Del Galdo et al., 2008). Using both *in vitro* and *in vivo* assays, we demonstrated that when the caveolin-1 scaffolding domain peptide (CSD, amino acids 82-101 of caveolin-1) is introduced into cells, it mimics the function of full-length caveolin-1 and thereby reverses the pathological effects of caveolin-1 deficiency such as collagen overexpression by fibroblasts, the hypermigration of monocytes toward CXCL12 *in vitro* and into damaged lung tissue *in vivo*, and enhanced monocyte differentiation into fibrocytes (Tourkina et al., 2005, 2008, 2010, 2011). The fact that in fibrosis both fibroblasts and monocytes are deficient in caveolin-1 is intriguing because monocytes differentiate into fibrocytes which in turn differentiate into fibroblasts. Thus cells derived from circulating monocytes may participate in fibrosis in a variety of target organs. By targeting monocyte/fibrocyte recruitment and fibrocyte differentiation, CSD may serve as a useful treatment for fibrotic diseases in a wide range of tissues.

The role of the chemokine receptor CCR5 in pulmonary fibrosis is particularly striking. CCR5 KO mice are resistant to bleomycin-induced pulmonary fibrosis in terms of tissue morphology and of collagen and fibrocyte accumulation in the tissue (Ishida et al., 2007). Moreover, in reconstitution experiments KO bone marrow was sufficient to provide protection in otherwise wild type mice while wild type bone marrow was sufficient to provide sensitivity in otherwise KO mice (Ishida et al., 2007), strongly supporting the ideas that bone marrow-derived cells (i.e., fibrocytes) are important players in the development of fibrosis *in vivo* and that CCR5 is a key regulator of their behavior. The levels of CCR5 and its ligands present support the idea that these are also important players in SSc and other human fibrotic diseases. CCR5 ligands are present at high levels in the serum and in affected tissues in SSc patients (Bolster et al., 1997; Hasegawa et al., 1999; Codullo et al., 2011; Gambichler et al., 2011, 2012; Bandinelli et al., 2012). Levels of CCR5 and its ligands in bronchoalveolar lavage fluid from patients with idiopathic pulmonary fibrosis (IPF) are significantly elevated (Capelli et al., 2005). CCR5 has also been identified as an essential mediator in hepatic fibrosis (Seki et al., 2009; Berres et al., 2010; Nellen et al., 2012; Stock et al., 2013) through regulating macrophage and fibrocyte infiltration.

Recently, we found that systemic bleomycin delivery using subcutaneously implanted osmotic minipumps can produce a very useful mouse model for SSc in which fibrosis is observed in the skin, lungs, and a variety of other internal organs (Lee et al., 2014). In the current study, the ability of CSD to block the progression of skin fibrosis in this model has been examined with particular emphasis on the role of CCR5. We find that CSD blocks the induction by bleomycin of dermal fibrosis while also blocking the accumulation of fibrocytes, CCR5, and CCR5 ligands in the skin. The importance of these findings to human fibrotic disease was validated by the observations that fibrocytes, CCR5, and CCR5 ligands accumulate in the fibrotic skin of SSc patients and that CSD blocks the migration of SSc monocytes toward CCR5 ligands. Interestingly, we also observed that, in this model, bleomycin caused a major decrease in the thickness of the subcutaneous adipocyte layer and that this effect, like dermal thickening, was also reversed by CSD. To the best of our knowledge, this is the first report on the ability of CSD to block the progression of dermal fibrosis and restore the adipose tissue layer *in vivo*.

## Materials and methods

### Pump model for systemic bleomycin treatment

The following procedures were approved by the MUSC Institutional Animal Care and Use Committee. Ten-week old, male CD1 mice (Charles River Laboratories, Boston, MA) maintained under pathogen-free conditions were treated systemically with bleomycin (Teva Parenteral Medicines, Inc., Irvine, CA) using an approach we refer to as the Pump Model. In this model, osmotic minipumps (ALZET 1007D; DURECT Corporation, Cupertino, CA) containing either 100 μl saline vehicle or bleomycin (100 U/kg) designed to deliver their contents at 0.5 μ l/h for 7 days were implanted under isofluorane anesthesia under the loose skin on the back of the mice slightly posterior to the scapulae. Pumps were removed on day 10 as recommended by the manufacturer. Each experiment was performed using at least six mice per group. In experiments involving CSD, starting the day before pump implantation, mice were injected i.p. daily for 28 days with 100 μl of CSD peptide (concentration 0.1 mM) or of vehicle (10% DMSO in water). CSD was synthesized as a fusion protein to the C terminus of the Antennapedia Internalization Sequence as previously described (Tourkina et al., 2008).

Mice were routinely sacrificed by cervical dislocation under isoflurane anesthesia on day 28 or at earlier times as indicated. Under deep anesthesia, the rib cage was opened to expose the thoracic cavity. Mice to be analyzed by histology or immunohistology were systemically perfused via the left ventricle sequentially with PBS and buffered zinc formalin fixative (Z-Fix, Anatech, Battle Creek, MI). Lower back skin tissue near the implanted pump was then removed and embedded in paraffin. Sections (4 μm) were stained with hematoxylin and eosin (H&E) or Masson's trichrome or immunohistochemically as described below.

To isolate bone marrow cells for use in cell migration experiments, following PBS perfusion femurs and tibiae were carefully cleaned from the adherent soft tissue. The tip of each bone was removed with a rongeur, and the marrow was harvested by inserting a syringe needle (27-gauge) into one end of the bone, and flushing with Dulbecco's Modified Eagle Medium (DMEM). The bone marrow cells were then filtered through a 70-mm nylon mesh filter (BD Falcon, USA) and counted.

### Histopathological assessment of mouse skin fibrosis

Morphologic characteristics of skin sections were assessed under a light microscope. The dermal thickness was defined as the distance from the top of the granular layer to the junction between the dermis and subcutaneous fat and was measured by a blinded observer.

### Human blood and skin donors

Under a protocol approved by the Institutional Review Board for Human Research, patients with SSc and interstitial lung disease (ILD) were recruited from the Scleroderma Clinic at the Medical University of South Carolina (MUSC). All patients fulfilled the American College of Rheumatology (formerly the American Rheumatism Association) criteria for SSc (Subcommittee, 1980). A written consent was obtained from all studied subjects.

Demographic and clinical characteristics of human subjects are summarized in Tables 1, 2. Systemic sclerosis patients were classified as having either limited cutaneous (lcSSc) or diffuse cutaneous (dcSSc) systemic sclerosis according to the criteria proposed by LeRoy et al. (1988). Disease duration was determined based on when the first non-Raynaud phenomenon symptoms were documented. The following criteria were used to define visceral involvement:

**Table 1:** **Clinical features of SSc patients and control subjects involved in monocyte experiments**.

		**SSc (***n*** = **11**)**	**Controls (***n*** = **20**)**
Age	Mean ± *SD* (years)	54.2 ± 10.9	40.7 ± 12.9
	Range (years)	37–69	18–55
Disease duration	Mean ± *SD* (years)	5.7 ± 5.3	
	Range (years)	1–16	
		**Number (%)**	**Number (%)**
Race	Caucasian (male)	2 (18.2)	3 (15.0)
	Caucasian (female)	4 (36.4)	7 (35.0)
	African-American (male)	3 (27.3)	2 (10.0)
	African-American (female)	3 (27.3)	8 (40.0)
Smoking status	Smoker	0 (0)	1 (5.0)
	Former smoker	1 (9.1)	1 (5.0)
Diagnosis	Limited cutaneous	6 (54.6)	
	Diffuse cutaneous	5 (45.5)	
	Overlap	1 (9.1)	
Symptoms	Pulmonary involvement (ILD)	7 (63.6)	
	Pulmonary hypertension	1 (9.1)	
	Gastrointestinal involvement	1 (9.1)	
	Cardiac involvement	2 (18.2)	
	Renal involvement	1 (9.1)	
	Raynaud phenomenon	11 (100)	
Autoantibodies	Antinuclear antibodies	11 (100)	
	Anti-topoisomerase I	4 (36.4)	
	Anti-centromere	0 (0)	

**Table 2:** **Clinical features of SSc patients providing skin biopsies**.

		**SSc (***n*** = **4**)**	**Controls (***n*** = **6**)**
Age	Mean ± *SD* (years)	62.0 ± 11.4	56.7 ± 12.4
	Range (years)	45–69	44–69
Disease duration	Mean ± *SD* (years)	4.7 ± 2.9	
	Range (years)	8–16	
		**Number (%)**	**Number (%)**
Race	Caucasian (male)	0 (0)	0 (0)
	Caucasian (female)	4 (100)	4 (66.7)
	African-American (male)	0 (0)	0 (0)
	African-American (female)	0 (0)	2 (33.3)
Smoking status	Smoker	0 (0)	0 (0)
	Former smoker	0 (0)	0 (0)

#### Pulmonary

Demonstration of abnormalities on high resolution computed tomographic (HRCT) scan (ground glass changes and/or fibrosis), pulmonary hypertension based on a right heart catheterization, restrictive changes on pulmonary function testing, or reduced diffusing capacity for carbon monoxide.

#### Gastrointestinal (GI)

History of gastro-esophageal reflux disease based on either subjective and/or objective findings. Some patients had symptoms of reflux requiring treatment with a proton pump inhibitor and/or H2-antagonist. Others had abnormal motility documented by esophageal manometry or findings of esophagitis on upper endoscopy.

#### Cardiac

Evidence on echocardiogram of left ventricular diastolic dysfunction, a pericardial effusion, elevated peak right ventricular systolic pressure, right ventricular and/or right atrial dilatation. Conduction abnormalities on a 12—lead EKG was also considered sufficient.

#### Renal

History of rapidly progressive renal failure.

#### Autoantibodies

Antinuclear antibodies (ANA) and anti-centromere antibodies were determined by immunofluorescent analysis on Hep-2 cell substrates. Anti-Scl-70 (topoisomerase I) antibodies were determined by enzyme immunoassay.

### Histopathology of human skin

Skin biopsy tissue from eight human subjects (four patients with SSc and four healthy controls) were fixed withbuffered zinc formalin (Z-Fix, Anatech, Battle Creek, MI), paraffin embedded and sectioned (4 μm), and stained with H&E or Masson's trichrome or immunohistochemically as described below.

### Human monocyte isolation

Human monocytes were isolated using standard protocols (Tourkina et al., 2010, 2011). Following centrifugation on density 1.083 Histopaque cushions to isolate peripheral blood mononuclear cell (PBMC), monocytes were then isolated from the PBMC by immunodepletion using a Dynal Monocyte Negative Isolation Kit (Invitrogen, Carlsbad, CA) resulting in a cell population of 95% Mac-1+ monocytes.

### Monocyte migration

Monocytemigrationassays were performed as previously described (Tourkina et al., 2011). Briefly, chemokine ligands (100 nM of MIP1α or MIP1β in RPMI 1640 with 1% BSA) were placed into the lower wells of Neuro Probe Multiwell Chemotaxis Chambers (Neuro Probe, Gaithersburg, MD) fitted with 5 μm pore size polycarbonate filters. 25 μ l of cell suspension (1 × 10^6^ cells/ml) with or without TGF-β pretreatment (45 min, 10 ng/ml in RPMI 1640 with 1% BSA) was placed in the upper wells. CSD peptide (0.1 μM) or control peptide was added to cells before they were placed in the upper chamber. After incubation for 2.5 h at 37°C in a 5% CO_2_ incubator, the filters separating the upper and lower wells were removed, fixed, and stained with DAPI. Cells on the underside of the membrane were counted in six high power (400×) fields per filter.

### Immunohistochemistry

Immunofluorescent staining of mouse and human skin tissue sections was performed (Tourkina et al., 2008) using routine methods. Fluorescent images were acquired using a Zeiss Axio Imager M2 fluorescence microscopy (Carl Zeiss, Germany).

### Statistical analyses

Numerical data are expressed as average ± s.e.m. and were analyzed using ANOVA with *post-hoc* Tukey's test to evaluate statistical significance. In all figures, statistical significance is expressed as ^*^p < 0.05, ^**^p < 0.01, and ^***^p < 0.001.

### Antibodies and chemokines used

eBioscience (San Diego, CA): Mouse anti-human CD45 (14-0459). Santa Cruz Biotechnology (Dallas, TX): Rabbit anti-caveolin-1 (sc-894). EMD Millipore (Billerica, MA): Rat anti-human procollagen I (MAB1912); Mouse anti-human procollagen I (MAB1912); Rabbit monoclonal anti-fatty acid binding protein 4 (FABP4) (MABS172). GTx Inc (Memphis, TN), anti-CCR5 (GTX61751). BD Biosciences (San Jose, CA): Rat anti-mouse CD45 (553076). Bioss Antibodies (Woburn, MA): Rabbit anti-MIP1α (bs-1045R); Rabbit anti-MIP1β (bs-2475R). In some cases primary antibodies were directly labeled using an Alexa Fluor 647 Protein Labeling Kit (Invitrogen, Carlsbad, CA, A-20173) as described by the manufacturer. In other cases, appropriate fluorescent secondary antibodies were used. Nuclei were routinely stained using 4′, 6-diamidino-2-phenylindole (DAPI) (Invitrogen). All immunohistochemical experiments were performed at least three times with similar results. Chemokines were obtained from Peprotech (Rocky Hill, NJ): Human MIP1α (300-08); Human MIP1β (300-09).

## Results

### Validation of a bleomycin-induced murine model for skin fibrosis

**Figure 1:**
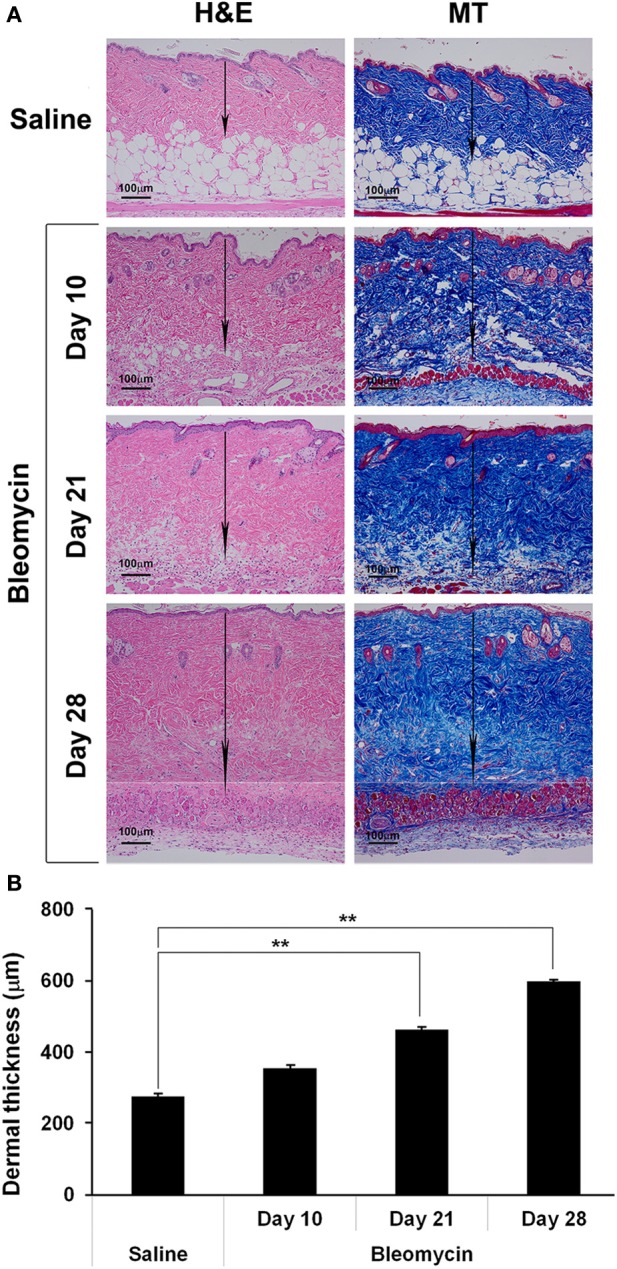
**Bleomycin-induced dermal fibrosis**. As described in the Methods, osmotic minipumps were used to deliver bleomycin or saline vehicle systemically to 10 week old male CD1 mice. Mice were sacrificed on days 10, 21, or 28. Lower back skin tissue near the implanted pump was harvested, paraffin sections cut and stained with H&E or Masson's trichrome (MT), and the thickness of the dermis measured (arrows). **(A)** Representative images. 100 μm bars are present in each panel. **(B)** Measurements (average ± s.e.m.) from six mice per category, six sites per mouse. Bleomycin induced significant dermal fibrosis as evidenced by increased dermal thickness and lipodystropy as evidenced by loss of the adipose cell layer.

We recently compared two methods of delivering bleomycin [Direct Model (directly into the lungs) and Pump Model (systemic delivery using implanted osmotic minipumps)]and found that the lung disease induced in the Pump Model was distinct from the disease induced in the Direct Model and more similar to the lung disease observed in SSc patients (Lee et al., 2014). Relevant to the current study, the Pump Model was also more similar to SSc in that dermal fibrosis was observed in the Pump Model (Figure 1), but not in the Direct Model (not shown). Dermal thickness showed a time-dependent increase in bleomycin-treated mice (Figure 1). By 10 days after treatment, a statistically significant increase in dermal thickness had occurred. By day 28 dermal thickness had increased to about 2-fold the level in control mice.

**Figure 2:**
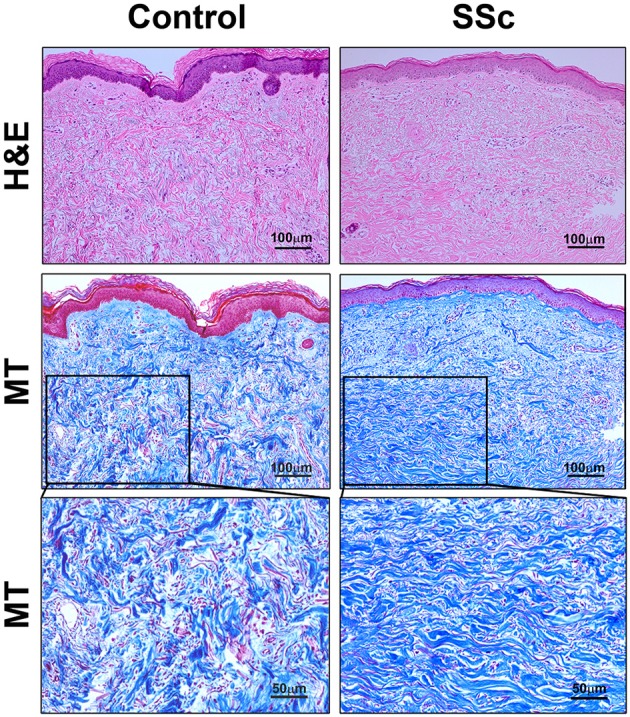
**Histopathology of skin lesions in SSc patients**. Skin sections from Control subjects and SSc patients were stained with H&E or Masson's Trichrome (MT). 100 μm bars are present in each low-magnification panel (top two rows). High-magnification MT images with 50 μm bars (third row) expand on the boxed regions in the low-magnification MT images. Note that collagen fibrils are packed more densely in SSc skin than in Control skin. Similar results were observed in four subjects in each category.

Fibrosis was also observed in the dermis of SSc patients. While the available tissue sections were not full-thickness and thus did not allow us to quantify the thickness of the dermis,images of SSc skin (particularly at higher magnification) revealed that collagen fibrils were packed more densely than in control skin (Figure 2). Interestingly, as in SSc patients (Marangoni et al., 2012), lipodystrophy (a loss of the subcutaneous adipose cell layer) was also observed in bleomycin-treated mice (Figure 1).

### CSD blocks bleomycin-induced dermal fibrosis and lipodystrophy

**Figure 3:**
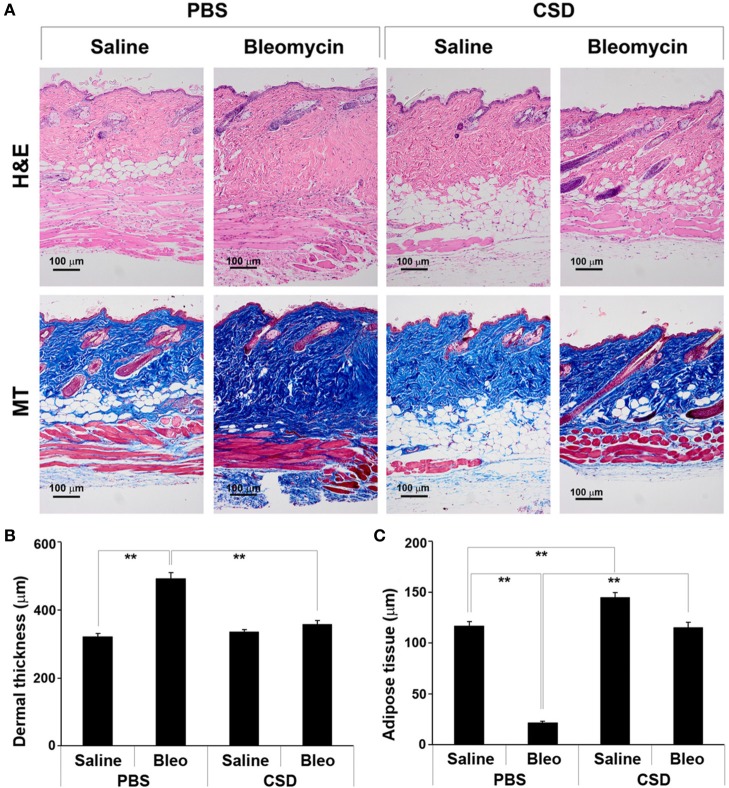
**CSD blocks dermal fibrosis and subcutaneous lipodystrophy**. As described in the Methods, osmotic minipumps were used to deliver bleomycin or saline vehicle systemically to 10-week old male CD1 mice. As indicated, mice also were injected daily i.p with 100 μl of CSD (concentration 0.1 mM) or of vehicle. Mice were sacrificed on day 28. Lower back skin tissue near the implanted pump was harvested, paraffin sections cut and stained with H&E or Masson's trichrome (MT), and the thickness of the dermis and adipose cell layer measured. **(A)** Representative images showing increased dermal thickness and thinning of the adipose cell layer in bleomycin-treated mice, both of which are blocked by CSD. Images of saline-treated Control mice show that CSD does not affect dermal thickness, but greatly increases the thickness of the adipose cell layer. 100 μm bars are present in each panel. **(B,C)** Measurements (average ± s.e.m.) from six mice per category, six sites per mouse. **(B)** Quantification of **(A)** for dermal thickness; **(C)** Quantification of **(A)** for thickness of the adipose cell layer.

We previously demonstrated that CSD can block bleomycin-induced lung fibrosis in the Direct Model (Tourkina et al., 2008). Here we demonstrate that CSD can also block bleomycin-induced skin fibrosis in the Pump Model (Figure 3). While CSD has no effect on dermal thickness in mice treated with saline vehicle, it almost completely blocks bleomycin-induced dermal thickening (Figures 3A,B). CSD also reversed the lipodystrophy induced by bleomycin treatment (Figures 3A,C). In addition, CSD caused a striking increase in the thickness of the adipose cell layer in control mice treated with saline vehicle.

### Increased accumulation of fibrocytes and cells positive for CCR5 and its ligands in fibrotic skin

**Figure 4:**
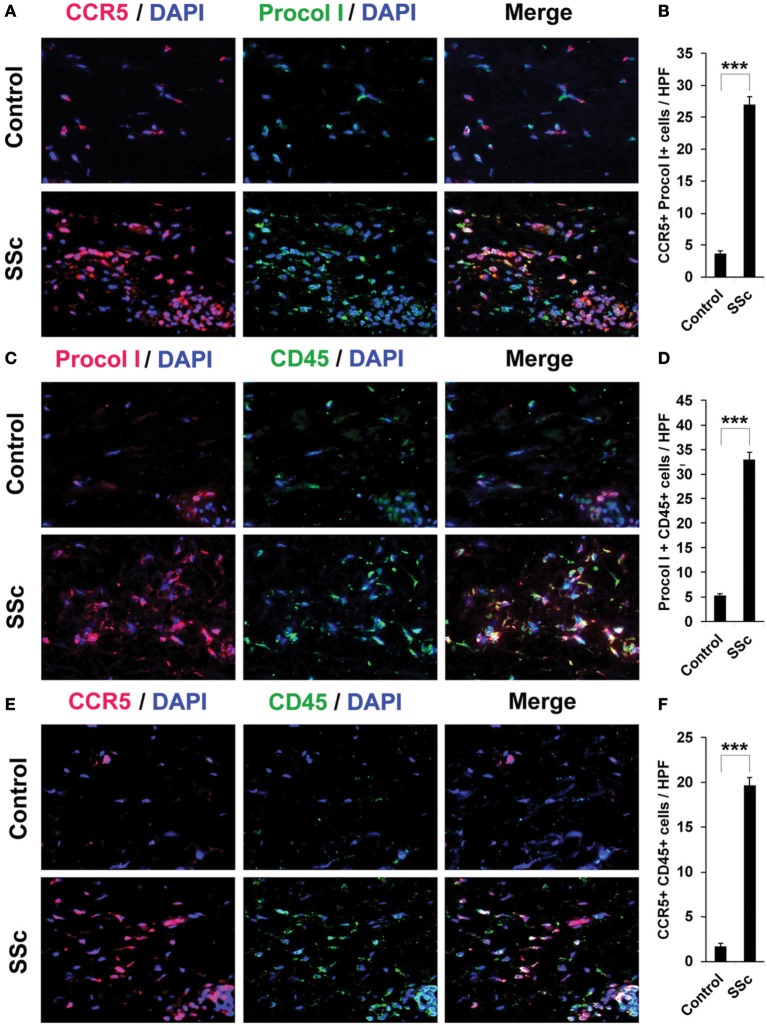
**Increased accumulation of fibrocytes and CCR5-positive cells in the dermis of SSc patients**. Fibrotic and control human skin sections were stained using the indicated antibodies and DAPI to detect nuclei. Large numbers of double-positive cells were observed in SSc dermis for each comparison (**A**, CCR5 vs. Procol I; **C**, Procol I vs. CD45; **E**, CCR5 vs. CD45), while almost none were observed in Control dermis. These representative fields were photographed at 400 × magnification. To quantify these observations, the number of double-positive cells was counted in three subjects per category, five high power fields (HPF) per subject. The data are presented in terms of the number of double-positive cells per HPF (average ± s.e.m.). **(B)** quantification of **(A)**; **(D)** quantification of **(C)**; **(F)** quantification of **(E)**.

**Figure 5:**
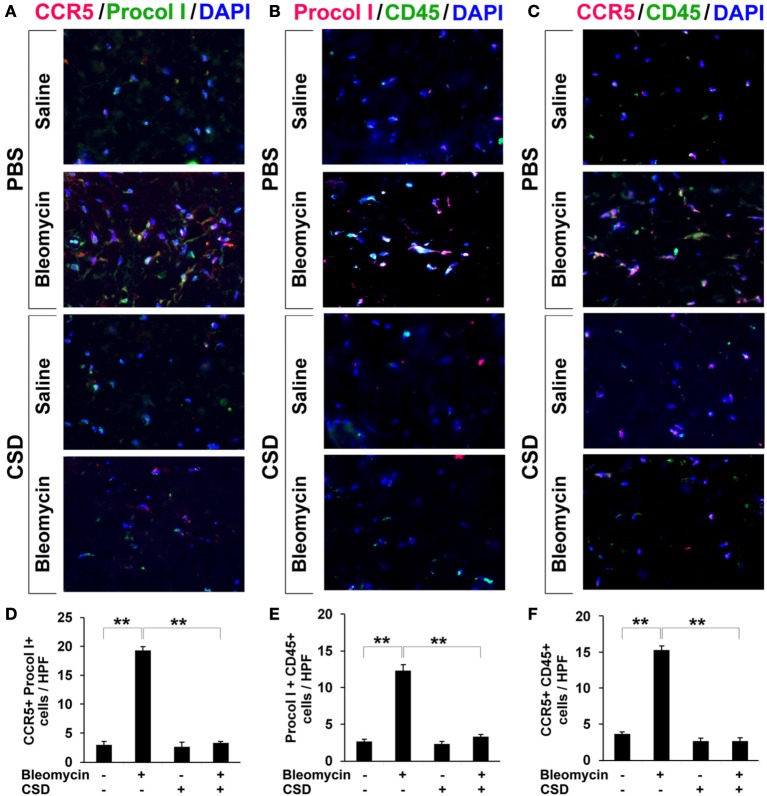
**Increased accumulation of fibrocytes and CCR5-positive cells in fibrotic mouse dermis is reversed by CSD**. Mice were treated with bleomycin and CSD as described in Figure 3. Skin sections were stained using the indicated antibodies and DAPI to detect nuclei. Large numbers of double-positive cells were observed in bleomycin-treated mice (that did not receive CSD) for each comparison (**A**, CCR5 vs. Procol I; **B**, Procol I vs. CD45; **C**, CCR5 vs. CD45), while almost none were observed in Control mice. CSD treatment almost completely blocked the accumulation of these cells. These representative fields were photographed at 400 × magnification. To quantify these observations, the number of double-positive cells was counted in three subjects per category, five HPF per subject. The data are presented in terms of the number of double-positive cells per HPF (average ± s.e.m.). **(D)** quantification of **(A)**; **(E)** quantification of **(B)**; **(F)** quantification of **(C)**.

To confirm that fibrocytes are important players in fibrosis, we determined fibrocyte levels in fibrotic human and mouse skin. CD45+/Procollagen I+ cells (i.e., fibrocytes) were detected at levels >5-fold higher in the fibrotic dermis of SSc patients (Figures 4C,D) and Pump Model mice (Figure 5B) than in control dermis where almost no fibrocytes were present. CCR5 has been shown to be a chemokine receptor of central importance in lung fibrosis (Ishida et al., 2007). To begin to determine the role of CCR5 in skin fibrosis, human and mouse skin sections were double-stained for CCR5 and either Procollagen I (Figures 4A,B) or CD45 (Figures 4E,F). Again, a similar >5-fold increase in double-positive cells was observed in all cases in fibrotic tissue, strongly suggesting that the fibrocytes present in fibrotic dermis are CCR5+.

**Figure 6:**
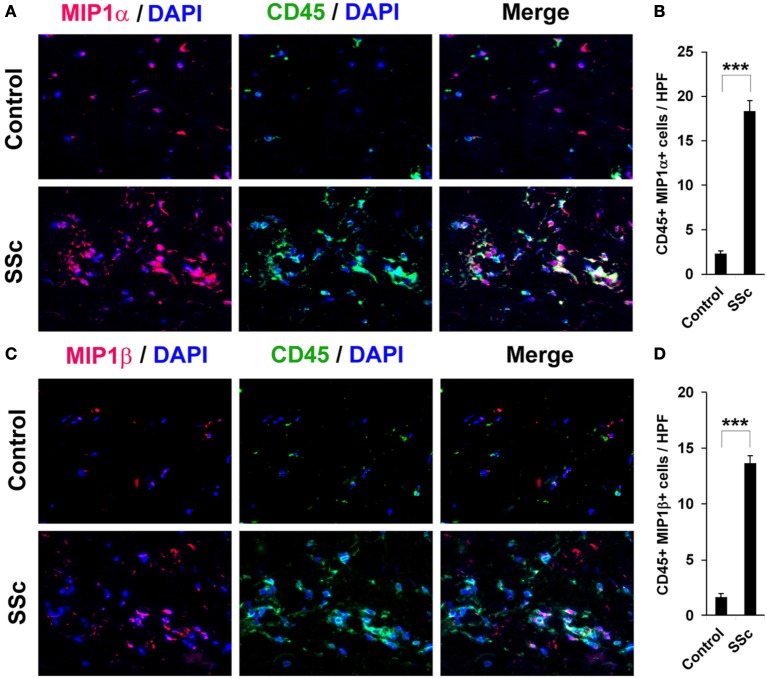
**Increased accumulation of CCR5 ligand-positive cells in the dermis of SSc patients**. Fibrotic and control human skin sections were stained using the indicated antibodies and DAPI to detect nuclei. Large numbers of double-positive cells were observed in SSc dermis for each comparison (**A**, MIP1α vs. CD45; **C**, MIP1β vs. CD45), while almost none were observed in Control dermis. These representative fields were photographed at 400 × magnification. To quantify these observations, the number of double-positive cells was counted in three subjects per category, five HPF per subject. The data are presented in terms of the number of double-positive cells per HPF (average ± s.e.m.). **(B)** quantification of **(A)**; **(D)** quantification of **(C)**.

**Figure 7:**
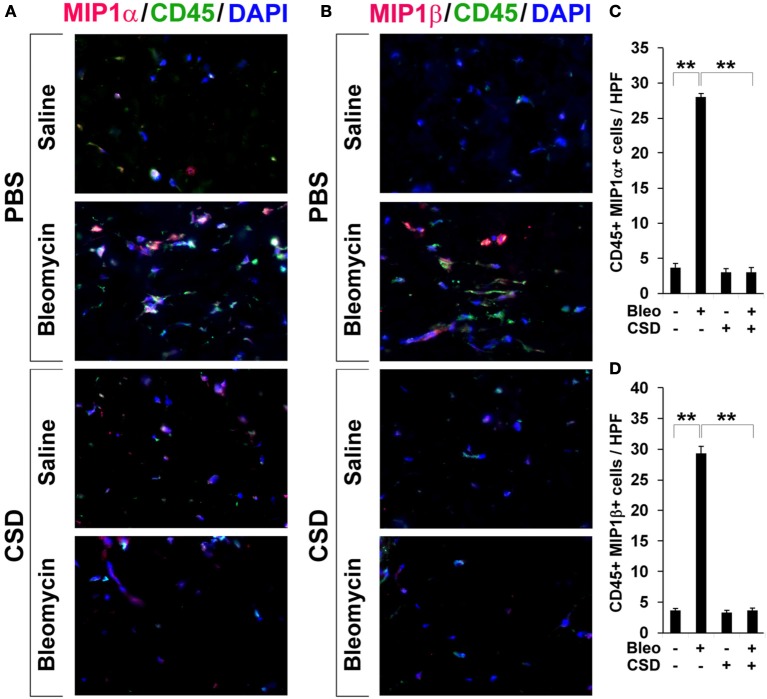
**Increased accumulation of CCR5 ligand-positive cells in fibrotic mouse dermis is reversed by CSD**. Mice were treated with bleomycin and CSD as described in Figure 3. Skin sections were stained using the indicated antibodies and DAPI to detect nuclei. Large numbers of double-positive cells were observed in bleomycin-treated mice (that did not receive CSD) for each comparison (**A**, MIP1α vs. CD45; **B**, MIP1β vs. CD45), while almost none were observed in Control mice. CSD treatment almost completely blocked the accumulation of these cells. These representative fields were photographed at 400 × magnification. To quantify these observations, the number of double-positive cells was counted in three subjects per category, five HPF per subject. The data are presented in terms of the number of double-positive cells per HPF (average ± s.e.m.). **(C)** quantification of **(A)**; **(D)** quantification of **(B)**.

To evaluate the accumulation in fibrotic skin of cells positive for CCR5 ligands, we double-stained human and mouse dermis for CD45 and for either MIP1α or MIP1β. CCR5 ligand-positive cells were detected at >5-fold higher levels in the fibrotic dermis of SSc patients (Figure 6) and Pump Model mice (Figure 7) than in control dermis where almost no double-positive cells were detected.

### CSD inhibits the accumulation of fibrocytes and cells expressing CCR5 and its ligands in fibrotic mouse dermis

In Figure 3, we showed that CSD inhibited dermal thickening in Pump Model mice. We next determined whether this beneficial effect is correlated with the reversal of the accumulation of fibrocytes and of cells expressing CCR5 and its ligands. In addition to inhibiting fibrosis and lipodystrophy (Figure 3), CSD also completely inhibited the accumulation of cells double-positive for Procollagen I and CD45, Procollagen I and CCR5, or CD45 and CCR5 (i.e., fibrocytes, Figure 5) and of cells double-positive for CCR5 ligands and CD45 (Figure 7). These results support the idea that the beneficial effect of CSD on dermal fibrosis may be due to the inhibition of the recruitment into the dermis of fibrocytes and other bone marrow-derived cells that express CCR5 and its ligands.

### CSD inhibits the migration toward CCR5 ligands of SSc monocytes

We next directly evaluated the ability of CSD to inhibit the recruitment of CCR5-expressing, bone marrow-derived cells. We previously showed that monocytes from SSc patients are deficient in caveolin-1, are hypermigratory toward the chemokine CXCL12, and overexpress its receptor CXCR4 (Tourkina et al., 2011). Treatment with CSD compensated for the caveolin-1 deficiency, thereby inhibiting CXCR4 overexpression and hypermigration toward CXCL12. The SSc monocyte phenotype (low caveolin-1, high CXCR4, hypermigration) could be induced in control monocytes by treatment with TGF-β.

**Figure 8:**
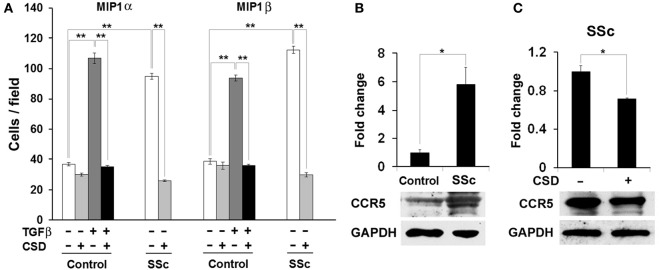
**The hypermigration of SSc monocytes and TGF-β-treated Control monocytes toward CCR5 ligands is regulated by caveolin-1. (A)** Migration experiments were performed as described in the Methods using Control or SSc monocytes. Cells were treated with TGF-β and CSD as indicated. Lower wells contained 100 nM of MIP1α or MIP1β. Cells that had migrated were counted in six high power fields per filter. Note that for both MIP1α and MIP1β, the induction of migration was extremely enhanced in both SSc monocytes and TGF-β-treated Control monocytes compared to untreated Control monocytes and that in both cases CSD completely blocked the enhanced migration. The data represent the average ± s.e.m. from four independent experiments using cells from different subjects. **(B)** CCR5 level is increased in SSc monocytes; **(C)** CSD decreases the level of CCR5 in SSc monocytes. Control and SSc monocytes were isolated as described in the Methods. SSc monocytes were treated with CSD as previously described (Tourkina et al., 2010). For Western blotting experiments, cells were extracted with boiling SDS-PAGE Sample Buffer. In **(B)**, the Western blot of a typical experiment shows a large increase in the level of CCR5 in SSc monocytes compared to Control monocytes and no change in the level of the loading control GAPDH. In **(C)**, the Western blot of a typical experiment shows that CSD treatment decreases the level of CCR5 in SSc monocytes without affecting GAPDH. The densitometric quantifications in **(B,C)** show the average ± s.e.m. in arbitrary units for four repeats of each experiment performed with cells from different subjects. The Control value in **(B)** and the CSD minus value in **(C)** are set to 1 arbitrary unit.

Analogous to our previous studies, we find that SSc monocytes accumulate high levels of CCR5, are hypermigratory toward the CCR5 ligands MIP1α and MIP1β, that the accumulation of CCR5 is inhibited by CSD, and that the hypermigration is totally blocked by CSD (Figure 8). Similarly, as in our previous studies, when control monocytes are activated *in vitro* using TGF-β, they become hypermigratory toward CCR5 and this migration is blocked by CSD (Figure 8). Thus, both monocytes activated *in vivo* (SSc) and monocyte activated *in vitro* (TGF-β) are hypermigratory toward CCR5 ligands and this migration is blocked by CSD.

**Figure 9:**
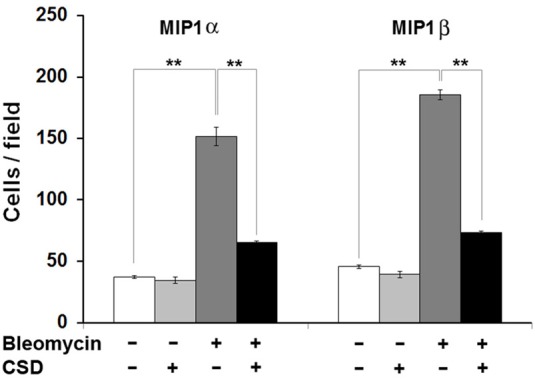
**Bone marrow cells from bleomycin-treated mice are hypermigratory toward CCR5 ligands and this migration is blocked by CSD**. Migration experiments were performed as described in the Methods using bone marrow cells from mice treated with bleomycin or saline vehicle. Mice were treated with CSD as indicated. Lower wells contained 100 ng/ml of MIP1α or MIP1β. Cells that had migrated were counted in six high power fields per filter. Note for both MIP1α and MIP1β, that the induction of migration was extremely enhanced in bone marrow cells from bleomycin-treated mice compared to bone marrow cells from vehicle-treated mice and that CSD completely blocked the enhanced migration. The data represent the average ± s.e.m. from three independent experiments using cells from different subjects.

Given that human monocytes from fibrotic subjects are hyper-migratory toward CCR5 ligands MIP1α and MIP1β and that this migration is blocked by CSD, we returned to the Pump Model to determine whether cell migration is also linked to fibrosis and caveolin-1 in mice. Indeed, bone marrow cells from bleomycin-treated mice were hypermigratory toward both MIP1α and MIP1β, and CSD delivered by i.p. injection *in vivo* knocked their levels of migration down to the Control mouse levels (Figure 9). CSD knocked down migration whether it was administered *in vitro* to bone marrow cells after their isolation from bleomycin-treated mice (not shown) or whether it was administered *in vivo* to bleomycin-treated mice prior to the isolation of bone marrow cells (Figure 9). These observations strongly support the idea that CSD inhibits dermal fibrosis by inhibiting monocyte/fibrocyte recruitment.

### CSD promotes the differentiation of subcutaneous adipose cells

**Figure 10:**
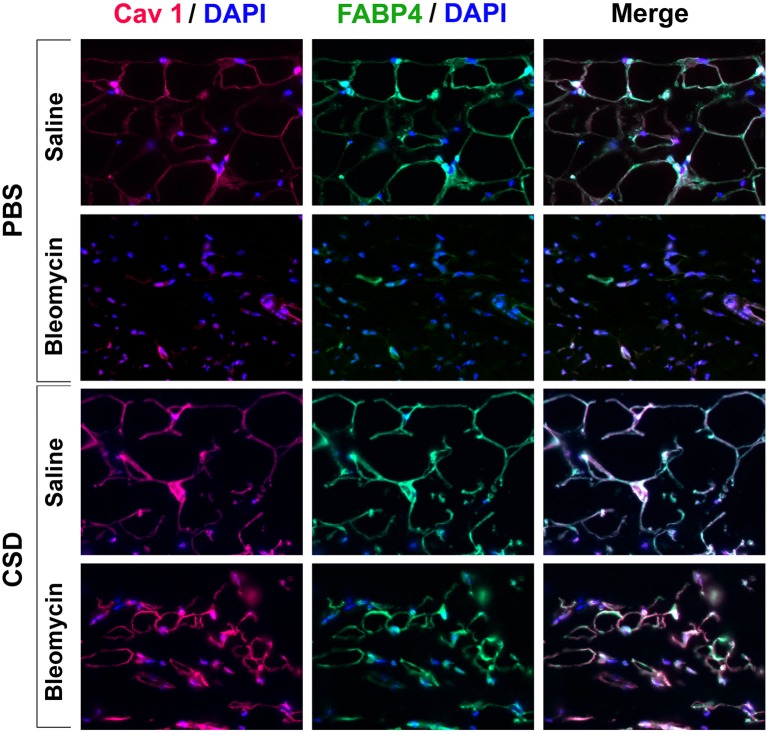
**FABP4/Caveolin-1 double staining of the adipocyte layer of mice treated with bleomycin and CSD**. Sections from the indicated mice were stained using the indicated antibodies and DAPI to detect nuclei. Staining at the level of the adipocyte layer is shown. These representative fields were photographed at 400 × magnification. Observations are described in detail in the text. Similar results were obtained in three independent experiments using cells from different subjects.

Figure 3 demonstrates that CSD, in addition to blocking the thickening of the dermis in bleomycin-treated mice, inhibits the thinning of the adipose cell layer in these mice and promotes the thickening of the adipose cell layer in control mice that did not receive bleomycin. To further understand these effects, skin sections were double-stained with antibodies against the adipocyte marker FABP4 and with antibodies against caveolin-1 which in this context is effectively also an adipocyte marker (Figure 10). Control mice (saline pump/ PBS injection) showed punctate and partial ring adipocyte membrane staining for both FABP4 and caveolin-1 that almost perfectly coincided. When saline pump mice received CSD injections, FABP4 and caveolin-1 staining were both enhanced, appearing as bright, ring staining of the entire adipocyte membrane. In bleomycin pump mice receiving PBS injections, in accord with the fact that by histological staining there was essentially no adipose cell layer, there was also almost no FABP4 or caveolin-1 staining. Finally, in bleomycin pump mice receiving CSD, bright, coincident ring staining for FABP4 and caveolin-1 was again observed. However, the diameter of these adipocytes was less than half the diameter of adipocytes in mice that did not receive bleomycin, strongly suggesting that these are immature adipocytes that are in the process of filling the void in the adipose cell layer created by bleomycin treatment. In any case, the effects of CSD on mice that received bleomycin and mice that did not receive bleomycin suggest that CSD promotes adipocyte differentiation *in vivo*.

## Discussion

In the current study, we have combined a mouse model for fibrosis that we have recently characterized (Lee et al., 2014) that closely models human SSc with our ongoing work on fibrocytes (CD45+, Collagen+ cells) in fibrosis and on caveolin-1 in fibrosis. In this model (Pump Model) fibrosis is observed in the skin and internal organs in addition to the lungs. We report here that treatment of Pump Model mice with CSD inhibits dermal fibrosis and at the same time inhibits the accumulation in the dermis of CD45+ cells expressing collagen, CCR5, and the CCR5 ligands MIP1α and MIPβ. Strongly supporting the idea that the beneficial effect of CSD results from the inhibition of the recruitment of bone marrow-derived cells into the dermis, we demonstrate that the migration of bone marrow cells toward CCR5 ligands is greatly enhanced in Pump Model mice receiving bleomycin to induce fibrosis compared to vehicle controls and that this enhanced migration is almost completely blocked in CSD-treated mice. The relevance of these observations to human disease is validated by the observations that CD45+ cells expressing collagen, CCR5, and CCR5 ligands are also present at high levels in the dermis of SSc patients, that SSc monocytes overexpress CCR5, and that the hypermigration of SSc monocytes toward CCR5 ligands is blocked by CSD treatment. Strongly supporting the idea that the beneficial effect of CSD results from the inhibition of the recruitment of bone marrow-derived cells into the dermis, we have recently observed (manuscript in preparation) that the migration toward several different chemokines of Pump Model mouse bone marrow cells is greatly enhanced compared to control mice bone marrow cells and that this enhanced migration is completely blocked when mice are treated with CSD in addition to bleomycin. Finally, we made the unexpected observation that in Pump Model mice dermal thickening is accompanied by thinning of the subcutaneous adipocyte cell layer and that CSD treatment, in addition to inhibiting the thickening of the dermis, inhibits the thinning of the adipocyte cell layer.

Most work on CCR5 has been performed in the context of human immunodeficiency virus infection/acquired immunodeficiency syndrome (HIV/AIDS) research. CCR5 serves as a co-receptor for the entry of HIV into T cells (Steffens and Hope, 2004). People carrying the CCR5 Δ32 mutation express little if any CCR5 at the cell surface and are resistant to HIV infection and may also be protected against bubonic plague (Liu et al., 1996; Samson et al., 1996). Interestingly, another chemokine receptor, CXCR4, that has been implicated in the regulation of fibrosis also serves as an HIV co-receptor. We find that both CCR5 (this paper) and CXCR4 (Tourkina et al., 2010, 2011) are overexpressed on SSc monocytes leading to their hypermigration toward cognate ligands. In both cases, CSD blocks this hypermigration.

The internalization of chemokine receptors plays an important role in ligand-induced cellular responses. After internalization, receptors can either be recycled back to the plasma membrane to be re-exposed to ligands or are trafficked for degradation. In the case of TGF-β receptors, caveolae-mediated internalization results in rapid turnover, thereby inhibiting TGF-β signaling (Di Guglielmo et al., 2003). If a similar mechanism applies to CCR5 and CXCR4, then the low level of caveolin-1 in SSc monocytes may result in the overexpression of CCR5 and CXCR4 in SSc monocytes due to a deficit in turnover. In considering the molecular mechanism underlying the accumulation of CCR5 in scleroderma monocytes and its reversal by CSD, it is interesting to note that CCR5 is a G protein-coupled receptor (Oppermann, 2004) and that caveolin-1 is known to bind to and modulate the function of G proteins (Li et al., 1995). While the significance to the current study remains to be determined, it is also noteworthy that the level of CCR5 onSSc-ILD and IPF T cells is low compared to control T cells (Capelli et al., 2005; Boin et al., 2008).

While CCR5 and its ligands have been studied in the context of lung fibrosis (Ishida et al., 2007), to the best of our knowledge, this is the first study of CCR5 in skin fibrosis. Our results are consistent with those of Ishida et al. who showed that bleomycin-induced fibrosis, fibrocyte recruitment, and inflammatory cell recruitment in general were blocked both in CCR5 and MIP1α (also known as CCL3) KO mice. Thus inhibition of the CCR5 axis may be a universal treatment for fibrotic diseases.

Skin fibrosis in SSc is associated with loss of subcutaneous adipose tissue (lipodystrophy) (Marangoni et al., 2012). Similarly, we observed a loss of subcutaneous adipose tissue concomitant with dermal fibrosis in the Pump Model, both of which were blocked by CSD. These observations are consistent with the fact that caveolin-1 KO mice are lean with small adipocytes (Razani et al., 2002). The mechanisms underlying lipodystrophy and its relationship to fibrosis are not known, but may involve regulation by caveolin-1 of the ability of pluripotent cells to differentiate either into myofibroblasts or adipocytes. Whether these cells might be monocytes, fibrocytes, or stem cells is an open question. This issue should be of great current interest in the treatment of SSc because stem cell therapy is receiving intense scrutiny as a treatment for several diseases including SSc, diabetes, rheumatoid arthritis, and lupus, and, indeed, limited promising results have already been obtained (Mizuno, 2010; Tyndall, 2011). Also noteworthy in contemplating the relationship betweenlipodystrophy and fibrosis are the observations that the level of the adipocyte-produced cytokine adiponectin is reducedin the serum and skin of SSc patients and that adiponectin has anti-fibrotic effects including the inhibition of collagen expression by fibroblasts (Arakawa et al., 2011; Fang et al., 2012; Lakota et al., 2012).

Another interesting potential mechanism for the loss of adipocytes in fibrotic tissue is that the rigid matrix has a variety of negative effects on adipocytes (Sun et al., 2013). In any case, because the mix of cells in the adipose layer is complex and their functions are many (Rosen and Spiegelman, 2014), it is likely that the relationship between fibrosis and adipogenesis will be complicated and important to the understanding and treatment of a wide range of diseases including not only fibrotic diseases, but also, diabetes, obesity, and wound healing.

In summary, the current studies provide novel information on the regulation of dermal fibrosis by caveolin-1 via its effects on the expression and function of CCR5 on monocytes and fibrocytes, the recruitment of fibrocytes and/or their progenitors into the affected tissue, and the thickness of the adipose cell layer in fibrotic tissue. In addition, these studies further validate CSD as a potential treatment for fibrotic diseases of the skin and other organs.

## Author contributions

Rebecca Lee participated in study design, performing experiments, data interpretation, and manuscript preparation. Beth Perry, Jonathan Heywood, and Charles Reese participated in performing experiments. Michael Bonner participated in performing experiments and in editing the manuscript. Corey M. Hatfield participated in data interpretation. Richard M. Silver participated in editing the manuscript. Stanley Hoffman and Richard P. Visconti participated in study design, data interpretation, and editing the manuscript. Elena Tourkina participated in study design, data interpretation, manuscript preparation, and editing the manuscript. All authors read and approved the final manuscript.

## Financial support

This work was supported by grants: NIH NIAMS R01 AR062078, R03 AR056767, and K01 AR054143 and a grant from the Scleroderma Foundation (to Elena Tourkina); USARMY/USAMRAA W81XWH-11-1-0508 (to Stanley Hoffman); NIH NIAMS P60 AR049459 (Multidisciplinary Clinical Research Center) (to Richard M. Silver); and an NIH NCRR Construction Grant C06 RR015455. Elena Tourkina also received the Marta Max Award from the Scleroderma Foundation.

### Conflict of interest statement

While none of the authors have received any financial benefit from this work, Drs. Hoffman and Tourkina are the Inventors on a use patent (#8,058,227) issued to the Medical University of South Carolina on the caveolin-1 scaffolding domain peptide as a treatment for fibrotic diseases. Drs. Hoffman and Tourkina are also the founders of a company, FibroTherapeutics, Inc., for the purpose of developing a drug based on the caveolin-1 scaffolding domain peptide. The other authors declare that the research was conducted in the absence of any commercial or financial relationships that could be construed as a potential conflict of interest.
